# Design of Circular Composite Cylinders for Optimal Natural Frequencies

**DOI:** 10.3390/ma14123203

**Published:** 2021-06-10

**Authors:** Gokhan Serhat

**Affiliations:** Max Planck Institute for Intelligent Systems, Heisenbergstr. 3, 70569 Stuttgart, Germany; serhat@is.mpg.de

**Keywords:** composite cylinders, stiffness tailoring, lamination parameters, free vibration modes, eigenfrequency separation, optimization

## Abstract

This study concerns optimizing the eigenfrequencies of circular cylindrical laminates. The stiffness properties are described by lamination parameters to avoid potential solution dependency on the initial assumptions of the laminate configurations. In the lamination parameter plane, novel response contours are obtained for the first and second natural frequencies as well as their difference. The influence of cylinder length, radius, thickness, and boundary conditions on the responses is investigated. The lamination parameters yielding the maximum response values are determined, and the first two mode shapes are shown for the optimum points. The results demonstrate that the maximum fundamental frequency points of the laminated cylinders mostly lie at the inner lamination parameter domain, unlike the singly curved composite panels. In addition, the second eigenfrequency shows a nonconvex response surface containing multiple local maxima for several cases. Moreover, the frequency difference contours appear as highly irregular, which is unconventional for free vibration responses.

## 1. Introduction

Laminated composite materials are extensively used in the construction, aerospace, automotive, and marine industries. In addition to their high stiffness-to-weight ratio, such materials offer the possibility of tailoring the stiffness properties for specific applications. Hence, the design of engineering structures made of laminated composites has been widely addressed in the literature.

One particular group of laminated structures comprises cylinders, which are commonly utilized as the shells of aircraft and satellites. These structures are frequently designed to provide optimal natural frequencies to prevent resonances under expected dynamic operating conditions. In this context, the fundamental natural frequency has been prevalently selected as the design objective to be maximized. For instance, Nshanian and Pappas [1] maximized the fundamental frequency of symmetric angle-ply cylindrical laminates by optimizing ply angle variation through the thickness. Lam and Loy [2] investigated the influence of boundary conditions and fiber orientation on the fundamental frequency of thin orthotropic laminated cylindrical shells. Later, Shakeri et al. [3] utilized finite element analysis with genetic algorithms to optimize simply supported laminated cylinders for the maximum fundamental frequency. Similarly, Koide and Luersen [4] computed optimal stacking sequences that maximize the fundamental frequency of laminated composite cylinders using the ant colony algorithm. Recently, Miller and Ziemiański [5] used genetic algorithms and deep neural networks to optimize the stacking sequence of composite cylindrical shells for the maximum fundamental frequency. The fundamental frequency of the laminated cylinders was also maximized along with buckling load in several multi-objective optimization studies [6,7].

Another widespread approach is the maximization of the difference between the adjacent eigenfrequencies of the structure [8,9,10]. This technique facilitates decreasing dynamic response amplitudes when the excitation frequencies lie in between the separated modal frequencies. However, there are few studies that concern the application of this approach to composite cylinders. In a recent study, Miller and Ziemiański [11] maximized the eigenfrequency gaps between several modes of laminated composite cylinders using genetic algorithms and neural networks.

In laminate stacking sequence optimization, the optimal designs may depend on the initial assumptions regarding the number of layers and layer thicknesses. As a remedy, lamination parameters have been introduced to describe the overall stiffness properties with intermediate variables [12]. This technique was also shown to provide convex solutions for certain responses such as the fundamental frequency of plates [13,14,15]. Lamination parameters have been widely used for maximizing the fundamental frequency of flat [16,17,18] and curved [19,20] laminated panels, as well as their eigenfrequency separation [10,21]. In one study regarding composite cylinders, Trias et al. [22] used lamination parameters to maximize the fundamental frequency. However, that work only presented the stacking sequence results for the analyzed cases without providing information on the optimal lamination parameters. To the author’s knowledge, fundamental frequency and the separation between adjacent eigenfrequencies have not been investigated in the lamination parameter domain for composite cylinders.

This study addresses the eigenfrequency optimization of circular composite cylinders. The stiffness properties are described using lamination parameters, which are optimized as the design variables. The first (fundamental) eigenfrequency or the separation between the first and second eigenfrequencies are maximized as the objective functions. The response contours for the first and second natural frequencies of the composite cylinders as well as their difference are obtained in the lamination parameter plane for the first time. In many cases, the maximum fundamental frequency points occurred at the interior region of the lamination parameter domain, thus requiring layer angles of multiple absolute values in the stacking sequence retrieval. This finding demonstrates that the stiffness tailoring required to obtain optimal dynamic properties can be remarkably different for cylindrical shells compared to singly curved panels, which have been reported to possess maxima on the boundary of the feasible domain [19,20]. In addition, the response surface for the second natural frequency appeared to be nonconvex for certain combinations of geometrical parameters. Moreover, the frequency separation contours exhibited banded behavior, which is unusual for free vibration responses, although it has previously been observed in forced dynamic analyses [19,23]. The presented results give valuable insights on the optimization of stiffness properties of cylindrical shells for improving dynamic characteristics.

## 2. Materials and Methods

### 2.1. Laminated Cylindrical Shell

**Figure 1:**
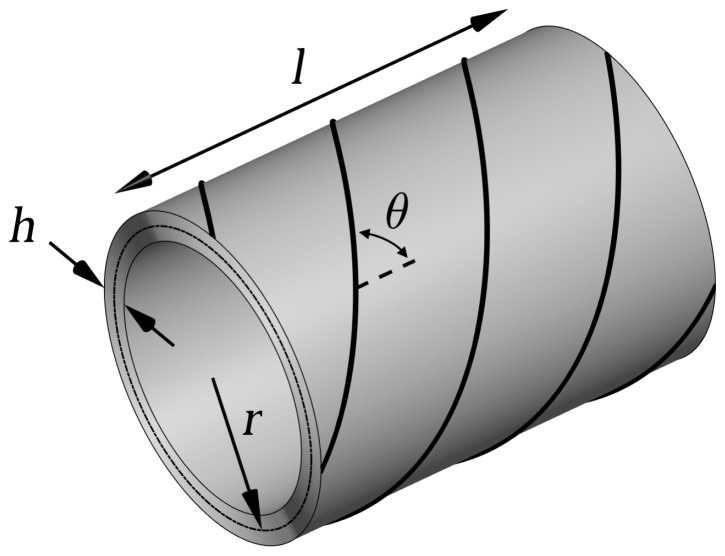
Laminated composite cylinder.

Simply supported and cantilever cylindrical laminates are considered in this study. Figure 1 shows the schematic of the analyzed circular composite cylinders having length *l*, radius *r*, thickness *h*, and fiber angle θ with respect to the longitudinal axis.

### 2.2. Stiffness Properties

The laminate stiffness properties are modeled using lamination parameters, which are the non-dimensional variables that govern the overall stiffness characteristics [24]. The considered laminates are assumed to consist of many homogeneously distributed balanced layers. Thus, the formulation requires two lamination parameters: $V_{1}$ and $V_{3}$ [25,26], which are defined as [27]

(1) $$\left\{\begin{array}{c} V_{1}\\ V_{3} \end{array}\right\}=\frac{1}{h}\sum_{k=1}^{N}t_{k}\left\{\begin{array}{c} \cos(2\theta_{k})\\ \cos(4\theta_{k}) \end{array}\right\}$$

where *N* is the number of layers in the laminate, $t_{k}$ are the layer thicknesses, and $\theta_{k}$ are the layer angles. The values of the lamination parameters are constrained by the following relations [25]:

(2) $$\begin{array}{c} -1\leq V_{1}\leq 1\\ \left(2V_{1}^{2}-1\right)\leq V_{3}\leq 1 \end{array}$$

Using the longitudinal modulus $E_{11}$, transverse modulus $E_{22}$, in-plane shear modulus $G_{12}$, and major Poisson’s ratio $\nu_{12}$, the material invariants ($U_{i}$) to be used within the formulation are defined as follows [28]:

(3) $$\left\{\begin{array}{c} U_{1}\\ U_{2}\\ U_{3}\\ U_{4}\\ U_{5} \end{array}\right\}=\left[\begin{array}{cccc} 3/8 & 3/8 & 1/4 & 1/2\\ 1/2 & -1/2 & 0 & 0\\ 1/8 & 1/8 & -1/4 & -1/2\\ 1/8 & 1/8 & 3/4 & -1/2\\ 1/8 & 1/8 & -1/4 & 1/2 \end{array}\right]\left\{\begin{array}{c} {E_{11}}^{2}/\left(E_{11}-E_{22}{\nu_{12}}^{2}\right)\\ E_{11}E_{22}/\left(E_{11}-E_{22}{\nu_{12}}^{2}\right)\\ E_{11}E_{22}\nu_{12}/\left(E_{11}-E_{22}{\nu_{12}}^{2}\right)\\ G_{12} \end{array}\right\}$$

In terms of lamination parameters and material invariants, the constitutive matrix relating in-plane strains to stresses can be expressed as [27]

(4) $$C_{p}=\left[\begin{array}{ccc} U_{1} & U_{4} & 0\\ U_{4} & U_{1} & 0\\ 0 & 0 & U_{5} \end{array}\right]+\left[\begin{array}{ccc} U_{2} & 0 & 0\\ 0 & -U_{2} & 0\\ 0 & 0 & 0 \end{array}\right]V_{1}+\left[\begin{array}{ccc} U_{3} & -U_{3} & 0\\ -U_{3} & U_{3} & 0\\ 0 & 0 & U_{3} \end{array}\right]V_{3}$$

The constitutive matrix for the transverse shear deformation can be stated as [28]

(5) $$C_{t}=\frac{5}{6}\left[\begin{array}{cc} G_{31}+\left(V_{1}+1\right)\left(G_{23}-G_{31}\right)/2 & 0\\ 0 & G_{23}+\left(V_{1}+1\right)\left(G_{31}-G_{23}\right)/2 \end{array}\right]$$

where $G_{23}$ and $G_{31}$ are the transverse shear moduli. The coefficient 5/6 is the shear correction factor, which equalizes the strain energies computed for the constant transverse stresses assumed with the first-order shear deformation theory and exact transverse stresses predicted by the three-dimensional elasticity theory [29].

The material properties of the graphite/epoxy laminae (Hercules AS/3501-6) used in the analyses are given in Table 1, where ρ denotes the density.

### 2.3. Finite Element Analysis

The solutions were computed through finite element analyses, which were performed using in-house tools developed with commercial software MATLAB (MathWorks, Natick, MA, USA). The cylinder was discretized with linear 4-node isoparametric shell elements having 3 translational and 2 rotational degrees of freedom at each node. This element is based on first-order shear deformation theory, and the details regarding its formulation can be found in [31].

Through elemental stiffness matrix generation, domain discretization, and assembly processes, nodal stiffness matrix *K* and nodal mass matrix *M* are obtained. One should note that previously derived constitutive matrices ($C_{p}$ and $C_{t}$) are used within the elemental stiffness matrices. Then, by using Newton’s second law, the equations of motion for undamped free vibrations can be written as

(6) $$Ku+M\ddot{u}=0$$

where *u* and $\ddot{u}$ are the nodal displacement and acceleration vectors, respectively.

An eigenvalue equation is obtained by assuming a solution in the form of $u=u_{n}e^{i\omega_{n}t}$ for Equation (6) [31]:

(7) $$(K-{\omega_{n}}^{2}M)u=0$$

Then, the natural frequencies of the panel ($\omega_{n}$) can be obtained by solving the following equation:

(8) $$\det(K-{\omega_{n}}^{2}M)=0$$

The smallest value of $\omega_{n}$ is the fundamental frequency denoted by $\omega_{1}$, and the second smallest frequency is $\omega_{2}$. The mode shape vectors ($u_{n}$) corresponding to the natural frequencies can be computed by solving Equation (7) for each $\omega_{n}$. In the present study, $\omega_{n}$ and $u_{n}$ were calculated using MATLAB’s “eigs” function. To present the eigenfrequencies independent from the particular dimensions, the frequency values in the main results were normalized using the following relation [22]:

(9) $${\bar{\omega }}_{n}=\omega_{n}r^{2}\sqrt{\rho /(E_{22}h^{2})}$$

### 2.4. Optimization

In the calculations, instead of only focusing on the optimal points, the response surfaces over the entire design space were investigated. Hence, full-factorial searches were performed using an approximate resolution of 0.1 for the lamination parameters. This approach ensures the complete exploration of the feasible domain with a total of 315 points.

## 3. Results and Discussion

This section covers the results of the free vibration analyses. The focus is on the investigation of the free vibration responses in the lamination parameter space, and the stacking-sequence retrieval is not addressed. In the literature, there are many studies that focus on finding the laminate configurations corresponding to the optimal lamination parameters [32,33,34].

### 3.1. Mesh Convergence and Validation

Prior to the principal investigations, convergence and validation studies were performed for a case taken from the reference [4], considering a simply supported composite cylinder having dimensions of l=0.2 m, r=0.1 m, h=8 mm. In the convergence analysis, the number of elements in the model was exponentially increased (∼1.5 times at each step) until the relative difference between subsequent solutions fell below 1%, ensuring a sufficient mesh resolution to obtain a precise solution. The calculations were performed for three cylinders with ($V_{1}$, $V_{3}$) pairs of (1.0, 1.0), (0.0, −1.0), and (−1.0, 1.0), which correspond to $0^{\circ }$, $\pm 45^{\circ }$, and $90^{\circ }$ laminates [35], respectively. By analyzing the results presented in Table 2, a model consisting of 832 elements was chosen for the investigations. In this model, there were 52 and 16 elements in circumferential and longitudinal directions, respectively. For the analysis of cylinders with different aspect ratios, the number of longitudinal elements was proportionally modified.

Next, the converged fundamental frequency values are compared to the ones presented in [4]. In the reference case, lamination parameters are not utilized but the analyzed cylinder consists of 64 layers, a relatively large number. Therefore, that case is suitable for comparison since the lamination parameter solution for homogeneous laminates converges to the solution for the corresponding layered composite with the increasing number of layers [18,21]. Table 3 shows the comparison of the fundamental frequencies. The relative difference magnitudes between the calculated values and the ones taken from the reference are below 0.5% for all three cases, validating the accuracy of the present model.

### 3.2. Eigenfrequency Optimization for Simply Supported Cylinders

**Figure 2:**
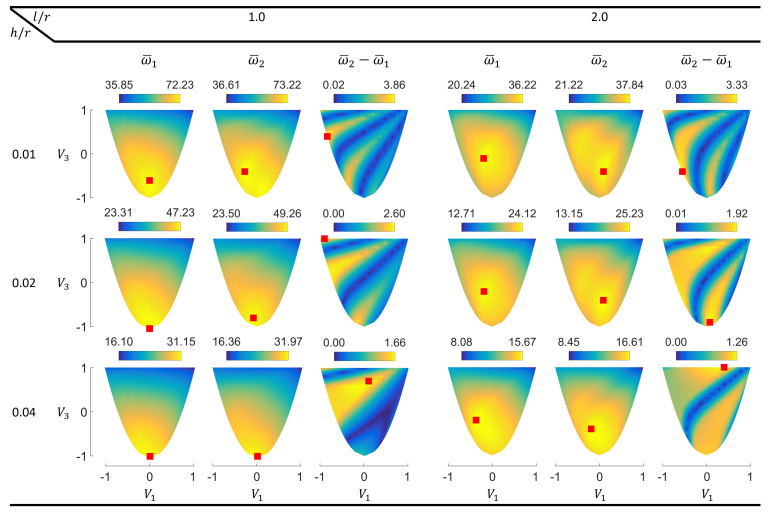
Lamination parameter plane contours of the first two normalized eigenfrequencies and their differences for simply supported circular composite cylinders.

This subsection covers the main results for the cylinders having simply supported boundary conditions, which are frequently used to model panel-like regions between the stiffeners of fuselages [36,37]. Figure 2 shows the contours of the first two eigenfrequencies (${\bar{\omega }}_{1}$ and ${\bar{\omega }}_{2}$) and their differences (${\bar{\omega }}_{2}-{\bar{\omega }}_{1}$) in the lamination parameter plane. The points providing the maximum response values are indicated by red squares.

The contour plots reveal several interesting points regarding the dynamic characteristics of cylindrical laminates. One behavior that stands out is the occurrence of the maximum fundamental frequency points at the interior region of the lamination parameter domain in several cases. Such inner points require layer angles of multiple absolute values in the stacking-sequence retrieval process [38]. Therefore, stiffness tailoring required to obtain optimal dynamic properties can be remarkably different for cylindrical shells compared to singly curved panels, which have been reported to possess maxima on the boundary of the feasible domain [19,20]. This finding also shows that searching only the boundary of the lamination parameters’ feasible domain is not always sufficient to obtain the optimal design points for the cylinders, although such an approach has been proposed to improve efficiency in the design of composite plates [39]. Sharp changes in certain regions of the fundamental frequency contours indicate the mode switching phenomenon, which has also been shown in the previous studies concerning composite panels [19,20,40].

Another noteworthy result is the nonconvex response surface of the second natural frequency, which shows multiple local maxima for l/r=2.0 and h/r=0.01,0.02. With the increasing thickness, the local peaks approach each other and look almost merged at h/r=0.04.

The frequency separation responses appear very irregular. The contours exhibit a banded character, which is unusual for free vibration responses although it has been previously observed in forced dynamic analyses [19,23]. Such behavior originates from the presence of modes with very close frequencies at certain design points. These modes do not have identical frequencies and shapes like the conjugate (or double) modes that have been reported to occur in cylindrical shells [41]. Hence, the lower colorbar limits appearing as “0.00” (due to the data presentation with two decimal places) do not correspond exactly to zero. Unlike maximum ${\bar{\omega }}_{1}$ and ${\bar{\omega }}_{2}$ points, many maximum ${\bar{\omega }}_{2}-{\bar{\omega }}_{1}$ points occur on the boundary. The points on the lower boundary are obtainable using angle ply laminates, where top boundary points require different cross ply laminates [25]. The multi-modal behavior observed in the second natural frequency and frequency difference contours indicate that the local search approaches prevalently used with the lamination parameters (e.g., direct search methods [16] or gradient-based algorithms [42,43,44]) may lead to locally optimal solutions when utilized with such responses.

**Figure 3:**
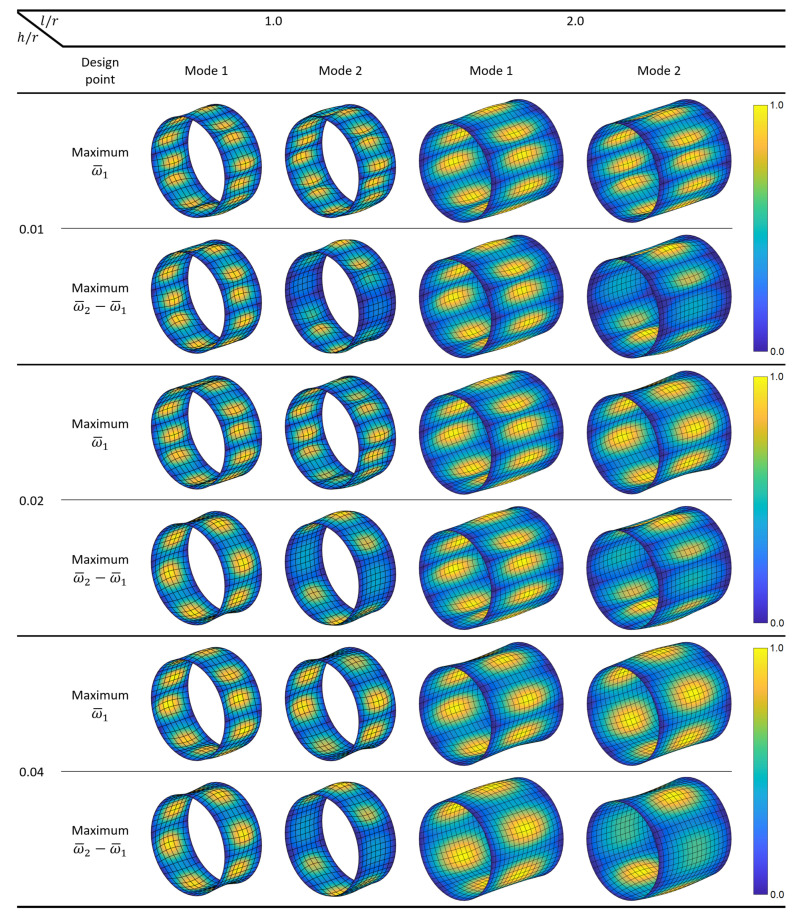
The first two mode shapes of simply supported circular composite cylinders optimized for maximum ${\bar{\omega }}_{1}$ and ${\bar{\omega }}_{2}-{\bar{\omega }}_{1}$.

Next, the shapes of the first two modes are investigated for the cylinders optimized for maximum ${\bar{\omega }}_{1}$ and maximum ${\bar{\omega }}_{2}-{\bar{\omega }}_{1}$. Figure 3 shows the mode shapes, where the colors indicate relative magnitudes of the resultant displacements within each mode. The results indicate that the first mode shape can change significantly for different combinations of the problem variables, and the second mode shape may contain fewer waves compared to the first mode shape. Such effects occur due to the complex dynamic properties of the cylindrical shells, whose fundamental natural frequency is not necessarily associated with the lowest wave index and natural frequencies do not fall in ascending order of the wave index [45]. The first mode shapes are different in the analyzed cases with l/r=1.0, where the second mode shapes are different in all cases.

### 3.3. Eigenfrequency Optimization for Cantilever Cylinders

**Figure 4:**
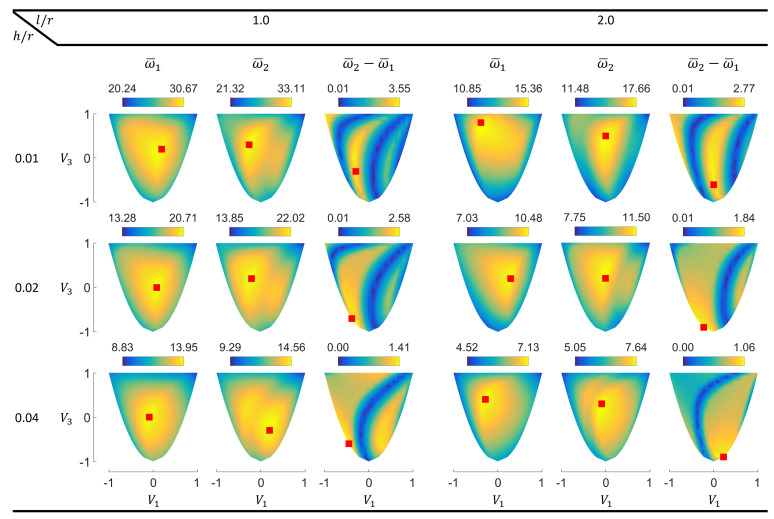
Lamination parameter plane contours of the first two normalized eigenfrequencies and their differences for cantilever circular composite cylinders.

The results for cantilever cylinders are presented in this subsection. Such structures can represent parts of aerospace structures such as space telescopes, hence their dynamic behavior is practically of interest [46]. Figure 4 shows the contour plots with the points of maximum values (red squares) for the first two normalized eigenfrequencies and their differences in the lamination parameter plane.

For the cantilever cylinders, all points resulting in the maximum first and second eigenfrequencies lie inside the feasible domain. For the lower cylinder aspect ratio (l/r=1.0), the maximum ${\bar{\omega }}_{1}$ points are in fact close to the middle point ($V_{1}=0.0$, $V_{3}=0.0$), which represents quasi-isotropic laminates [25].

The ${\bar{\omega }}_{2}$ contours for the cantilever cylinders also contain multiple local optima. However, unlike the simply supported panels, the local optima are also present for l/r=1.0. More differently, the local peaks for this aspect ratio get further apart and become clearer with the increasing thickness.

In the frequency difference contours, the irregular responses consisting of ridges and valleys are observed for the cantilever cylinders as well. Similar to the simply supported panels, increasing the h/r ratio results in the shift and partial disappearance of the observed bands.

**Figure 5:**
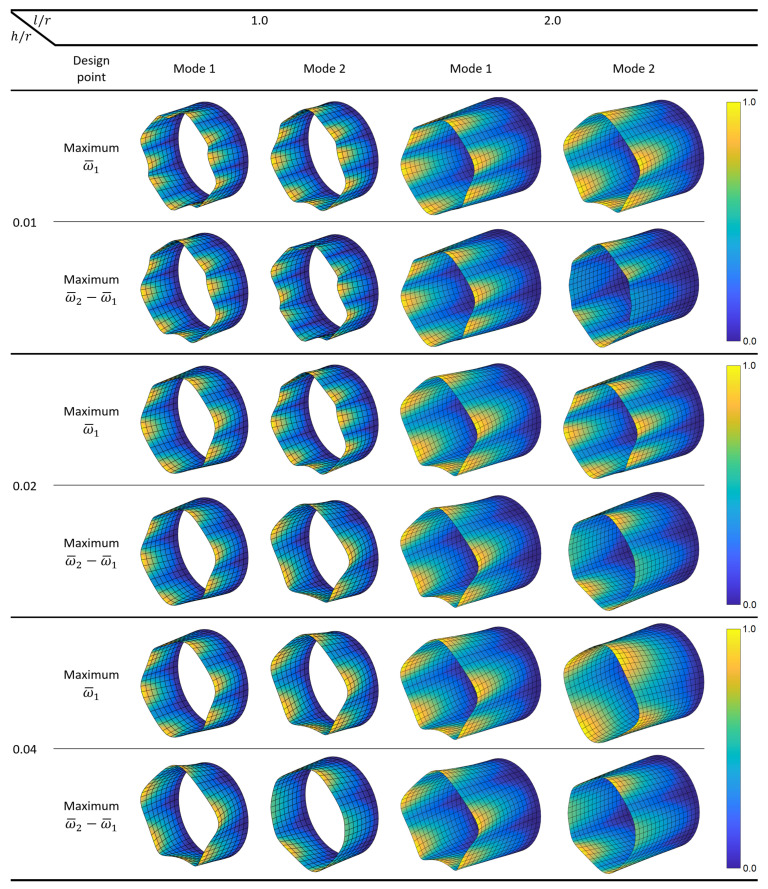
The first two mode shapes of cantilever circular composite cylinders optimized for maximum ${\bar{\omega }}_{1}$ and ${\bar{\omega }}_{2}-{\bar{\omega }}_{1}$.

Figure 5 shows the first and second mode shapes of cantilever circular composite cylinders optimized for maximum ${\bar{\omega }}_{1}$ and maximum ${\bar{\omega }}_{2}-{\bar{\omega }}_{1}$. Similar to the simply supported cylinders, no consistent patterns are observed between the number of circumferential waves and the order of the modes. In fact, for cylinders with l/r=2.0 and maximum ${\bar{\omega }}_{1}$, the first and second mode shapes interchange when h/r is increased from 0.01 to 0.02. Such behavior demonstrates the remarkable influence of the thickness on the modal dynamics. The number of circumferential waves in the modes generally decreases with the increasing thickness and l/r ratio conforming with the results presented in [46]. Once again, all second mode shapes are different for maximum ${\bar{\omega }}_{1}$ and ${\bar{\omega }}_{2}-{\bar{\omega }}_{1}$ designs.

Neither simply supported nor cantilever cylinders exhibit low-order mode shapes that involve significant average transverse displacements, which occur within the beam-like global bending modes of high-aspect-ratio structures. The lack of such modes with bulk transverse motion is due to the relatively low l/r proportion of the analyzed cylinders. Longer cylinders can manifest different dynamics due to the emergence of additional mode shapes, which have been demonstrated to change in a complicated fashion with the length of the shell [47].

## 4. Conclusions

In this study, the eigenfrequency optimization of circular laminated cylinders was addressed. The stiffness properties were expressed in terms of lamination parameters to prevent possible solution dependency on the initial assumptions of the laminate configuration and to give a broader insight into the influence of layer angles on dynamic responses. The contours for the first (fundamental) and second natural frequencies of the composite cylinders as well as their difference were obtained in the lamination parameter plane for the first time.

In the majority of analyzed cases, the maximum fundamental frequency points occurred at the interior region of the lamination parameter domain, thus requiring layer angles of multiple absolute values in the stacking sequence retrieval. This finding demonstrates that the stiffness tailoring required to obtain optimal dynamic properties can be remarkably different for cylindrical shells compared to singly curved panels, which have been reported to possess maxima on the boundary of the feasible domain.

The response surface of the second eigenfrequency appeared to be nonconvex for certain combinations of model parameters. In addition, the frequency separation contours exhibited a banded character, which is unusual for free vibration responses although it has previously been observed in forced dynamic analyses. Such behavior arises from the existence of modes with very close frequencies at certain design points. Increasing the laminate thickness caused the discovered bands to shift and partially vanish.

Due to the intricate dynamics of the cylindrical shells, no coherent relations were detected between the shape and order of the modes. This inconsistency has also been experimentally evidenced in the literature. In a particular case, the first two mode shapes even switched their order with the alteration of the thickness, despite both structures being optimized for the maximum fundamental frequency. Such an occurrence also manifests the distinctive influence of the laminate thickness on the free vibrations of the cylindrical shells. Moreover, increasing the thickness was shown to generally decrease the number of circumferential waves within the modes.

The presented results give valuable insights on tailoring the stiffness properties of composite cylindrical shells for improving dynamic characteristics. The analyzed simply supported and cantilever cylinders can be particularly related to the intermediate and end sections of circumferentially stiffened aerospace structures, respectively. In the design of such structures, optimization of the eigenfrequencies can facilitate avoiding resonances under operating conditions. However, one should note that the effectiveness of this technique depends on the type and frequency content of the actual dynamic forces. Forced vibration analyses can be performed to predict the functional performance for specific excitation sources.

In future studies, additional lamination parameters can be used in the formulation to explore a larger design space. The influence of other geometric values and boundary conditions on the results can also be investigated.

## Figures and Tables

**Table 1:** Material properties of unidirectional graphite/epoxy laminae [4,30].

$E_{11}$	128 GPa
$E_{22}$	11 GPa
$G_{12}=G_{13}$	4.48 GPa
$G_{23}$	1.53 GPa
$\nu_{12}$	0.25
ρ	1500 kg/m${}^{3}$

**Table 2:** Convergence analysis for the fundamental frequency. The frequency values are in Hz. Starting with the second-lowest number of elements, the percentage differences with respect to the previous values are given next to the frequency values followed by commas.

($V_{1}$,$V_{3}$)	Number of Elements				
	168	252	396	572	832
(1.0, 1.0)	1883.4	1850.9, 1.73%	1820.5, 1.64%	1805.1, 0.85%	1795.7, 0.52%
(0.0, −1.0)	3749.4	3632.2, 3.13%	3539.5, 2.55%	3489.8, 1.40%	3455.2, 0.99%
(−1.0, 1.0)	2120.0	2097.9, 1.04%	2079.6, 0.87%	2070.1, 0.46%	2063.9, 0.30%

**Table 3:** Comparison of the fundamental frequencies obtained in the present study and ref. [4].

Ref. [4]		Present		Difference in $f_{1}$
Stacking-Sequence	$f_{1}$ (Hz)	($V_{1}$, $V_{3}$)	$f_{1}$ (Hz)	
${\left[0_{32}\right]}_{s}$	1803.4	(1.0, 1.0)	1795.7	−0.43%
$\pm {\left[45_{16}\right]}_{s}$	3450.3	(0.0, −1.0)	3455.2	0.14%
${\left[90_{32}\right]}_{s}$	2061.2	(−1.0, 1.0)	2063.9	0.13%

## Data Availability

The data presented in this study are available on request from the author.
